# The place of trauma in the treatment of personality disorders

**DOI:** 10.3402/ejpt.v6.27629

**Published:** 2015-04-08

**Authors:** Theo J. M. Ingenhoven

**Affiliations:** Center for Psychotherapy, Pro Persona, Lunteren, The Netherlands

In DSM-5, the classification of posttraumatic stress disorder (PTSD) has broadened its scope. The stressor criterion is more explicit with regard to events that can be qualified as a traumatic experience. Moreover, the specifier “with dissociative symptoms” was introduced for individuals experiencing depersonalization or derealization. However, this extension does not fully cover the more profound symptomatic expressions and developmental trajectories of severe and lasting (childhood) abuse and neglect, nor their impact on the development of self and interpersonal functioning as described for personality disorders (as in the alternative DSM-5 model in Section III). During the last decades a number of concepts have been suggested to describe these more profound phenomena: type II disorder (Terr, 1991), complex PTSD (Herman, 1992), enduring personality change after catastrophic experience (WHO, 1992), PTSD related to childhood abuse (Cloitre, Koenen, Cohen, & Han, 2002), complex posttraumatic self-dysregulation (Ford, Courtois, van der Hart, & Nijenhuis, 2005), disorders of extreme stress, not otherwise specified (DESNOS; van der Kolk, Roth, Pelcovitz,Sunday, & Spinazzola, 2005), and developmental trauma disorder (van der Kolk, 2005). However, from a developmental perspective, the etiological role of trauma in complex PTSD is anything but simple. Pre-trauma factors are significant (genetic vulnerability) and what happens after the trauma has the biggest impact (context)! Unfortunately, none of these concepts were incorporated within the new version of the official APA classification system.

With respect to the treatment of PTSD, cognitive behavioral therapies currently dominate the field of treatment approaches. Imaginary exposure and Eye Movement Desensitization and Reprocessing (EMDR) are straightforward evidence-based interventions, widely available to diminish symptoms like intrusive distressing memories, hyper arousal, flash backs, dreams, and nightmares. The aim is to reduce these symptoms within a short period of time.

The majority of PTSD patients (Type I) are characterized by failed prefrontal inhibition of limbic activity. In contrast, however, in the dissociative subtype (feeling zoned out; detached from body) PTSD high prefrontal activation in conjunction with inhibited limbic activation was found.

In complex PTSD, clinicians and patients struggle with long-standing and multifaceted problems (especially suffering from personality disorders, dissociative disorders, mood disorders, and somatic symptom-related disorders), more eclectic approaches are needed integrating emotion regulation and interpersonal functioning strategies as well as psychodynamic understanding. Especially when childhood sexual or physical abuse was long lasting, repetitive, and induced by attachment figures, and when emotional support was lacking, the impact on personality development can be so devastating that other treatment efforts are necessary to establish a trustful therapeutic relation as a starting point for cautious exploration. Evidence-based psychotherapeutic models for (borderline) personality disorders all describe efforts to treat PTSD symptoms within such a specific psychotherapeutic frame of reference. Most of these treatments are intensive (minimal once or twice a week) and long lasting (years). In dialectical behavior therapy (DBT), the initial phase of treatment explicitly focuses on stabilizing by diminishing para-suicidal and self-destructive behaviors, as well as treatment-interfering behaviors. Individual cognitive treatment is combined with skill training to improve emotion regulation. As soon as the patient is skilled and stabilized (most of the time after an initial first year of treatment), DBT will next focus on the treatment of PTSD, using cognitive behavioral approaches like imaginary exposure. During transference focused psychotherapy (TFP), in the mid-phase of treatment, the theme of abuse will be activated within the transference (split self and object representations centered around aggression and hatred). Identification with both victim and perpetrator is elaborated, and sexual and aggressive impulses should be disentangled, with the purpose of resolving inner conflicts, fostering identity integration, and enhancing adaptive functioning. Also in schema-focused therapy (SFT), the treatment of trauma and PTSD is not part of the initial phase of treatment. The first phase of treatment involves identifying maladaptive schemas and building up adaptive capacities (healthy adult mode). Enough social support is a prerequisite to focus on specific former traumatic experiences. SFT describes specific strategies for treating traumatic experiences, like experiential techniques and imagination with rescripting. In mentalization-based treatment (MBT), the concept of mentalizing is used to broaden the perspective on trauma treatment. Mentalizing goes offline when defensive (fight–flight–freeze) responses become activated, promoting rapid responses to imminent danger. In particular, the impact of “attachment trauma” on emotion regulation and mentalizing reflects a dual liability (extreme distress plus impaired development emotion regulation capacities). Later in life, trauma-triggered hyperactivation of the attachment system, and the failure to mentalize, induces primitive modes of thought: psychic equivalence mode, pretend mode, and teleological mode. According to the MBT treatment, model treatment involves far more than processing traumatic memories. Treatment helps the patient to help the self to mentalize trauma and relationship conflicts, in order to develop more secure attachments. The patient uses the therapist as a mirror to understand the self: a “surrogate prefrontal cortex (PFC)”; it provides a buffer between feeling and action: a “pause button”, an opportunity for the patient to reconstruct his or her narrative within a safe and containing environment (first priority), and grounded in reality. In this way, the therapist often becomes the “object of hope.”

What these evidence-based treatments for (borderline) personality disorder have in common is their clear contract setting, their supportive common factors, their efforts to first stabilize their patients before exposing them to traumatic memories, their focus on maintaining a trustworthy therapeutic relationship, and their efforts to tailor psychotherapeutic strategies toward specific vulnerabilities and capabilities to regulate emotions and control (self-destructive) impulses. So, in complex PTSS and personality disorders, the central therapeutic task is NOT specifically to work with the content of traumatic events, but rather involves supporting a mentalizing stance in relation to the meaning and effect of trauma. The focus is primarily on the patients’ mind, not on the event, on the process rather than the content. Over and beyond the holding environment of the therapeutic relation, phase specific and carefully tailored, symptom-focused approaches like exposure and EMDR can reduce typical PTSD symptoms.

## References

[CIT0001] Terr L. C (1991). Childhood traumas: an outline and overview. American Journal of psychiatry.

[CIT0002] Herman J. L (1992). Complex PTSD: a syndrome in survivors of prolonged and repeated trauma. J Trauma Stress.

[CIT0003] WHO (1992). International Statistical Classification of diseases and Related Health problems.

[CIT0004] Cloitre M, Koenen K. C, Cohen L. R, Han H (2002). Skills training in affective and interpersonal regulation followed by exposure: A phase-based treatment for PTSD related to childhood abuse. Journal of Consulting and Child Psychology.

[CIT0005] Ford J. D, Courtois C. A, van der Hart O, Nijenhuis E. R. S (2005). Treatment of complex posttraumatic self-dysregulation. Journal of Traumatic Stress.

[CIT0006] van der Kolk B. A (2005). Developmental trauma disorder. towards a rational diagnosis for children with complex trauma histories. Psychiatric Annals.

[CIT0007] van der Kolk B. A, Roth S, Pelcovitz D, Sunday S, Spinazzola J (2005). Disorders of extreme stress: The empirical foundation of a complex adaptation to trauma. Journal of Traumatic Stress.

